# Anodic Electrodeposition of Chitosan–AgNP Composites Using In Situ Coordination with Copper Ions

**DOI:** 10.3390/ma14112754

**Published:** 2021-05-23

**Authors:** Dmitry S. Kharitonov, Aliaksandr A. Kasach, Agnieszka Gibala, Małgorzata Zimowska, Irina I. Kurilo, Angelika Wrzesińska, Lilianna Szyk-Warszyńska, Piotr Warszyński

**Affiliations:** 1 Jerzy Haber Institute of Catalysis and Surface Chemistry, Polish Academy of Sciences, Niezapominajek 8, 30-239 Krakow, Poland; ncgibala@cyf-kr.edu.pl (A.G.); nczimows@cyf-kr.edu.pl (M.Z.); ncszyk@cyf-kr.edu.pl (L.S.-W.); piotr.warszynski@ikifp.edu.pl (P.W.); 2 Department of Chemistry, Electrochemical Production Technology and Materials for Electronic Equipment, Chemical Technology and Engineering Faculty, Belarusian State Technological University, Sverdlova 13a, 220006 Minsk, Belarus; 3 Department of Molecular Medical Microbiology, Chair of Microbiology, Faculty of Medicine, Jagiellonian University Medical College, Czysta 18, 31-121 Krakow, Poland; 4 Department of Physical, Colloid and Analytical Chemistry, Organic Substances Technology Faculty, Belarusian State Technological University, Sverdlova 13a, 220006 Minsk, Belarus; kurilo@belstu.by; 5 Department of Molecular Physics, Faculty of Chemistry, Lodz University of Technology, 90-924 Lodz, Poland; angelika.wrzesinska@dokt.p.lodz.pl

**Keywords:** chitosan, copper, electrodeposition, composite, AgNPs, antibacterial properties

## Abstract

Chitosan is an attractive material for biomedical applications. A novel approach for the anodic electrodeposition of chitosan–AgNP composites using in situ coordination with copper ions is proposed in this work. The surface and cross-section morphology of the obtained coating with varying concentrations of AgNPs were evaluated by SEM, and surface functional groups were analyzed with FT-IR spectroscopy. The mechanism of the formation of the coating based on the chelation of Cu(II) ions with chitosan was discussed. The antibacterial activity of the coatings towards *Staphylococcus epidermidis* ATCC 35984/RP62A bacteria was analyzed using the live–dead approach. The presented results indicate that the obtained chitosan–AgNP-based films possess some limited anti-biofilm-forming properties and exhibit moderate antibacterial efficiency at high AgNP loads.

## 1. Introduction

Nowadays, natural polymers are of significant importance due to their unique properties and possibility of production from renewable sources. Chitosan (CS) is one of the most promising polysaccharide biopolymers with high practical potential due to its non-toxic nature, biocompatibility, antimicrobial activity, film-forming properties, relative chemical, and mechanical stability [1,2]. These properties make CS coatings useful for biomedical, packaging, pharmaceutical, and water-treatment applications [2,3,4,5,6]. Nevertheless, their usage in the biomedical sector remains dominant.

Numerous studies have investigated the antibacterial properties of CS [1,2,7,8,9]. The exact mechanism of the antibacterial activity of CS is not yet fully understood, but it is presumably connected to its polycationic structure in acidic aqueous solutions (pH < 6.3), that can interact with the negatively charged cell walls of bacteria, causing their disruption [10]. In the case of solid CS films, antibacterial activity is significantly reduced, which is connected to the smaller CS-bacteria contact area and pH, often above 6.3 [10,11]. Foster and Butt reported [12] that 20-µm-thick CS films showed no inhibitory effect against *E. coli*, *S. aureus*, or *S. epidermidis* ATCC 984/RP62A bacteria. On the other hand, the high antibacterial activity was reported for CS nanoparticles and connected to their large surface area and high affinity with bacterial cells [13].

To improve antibacterial functionality, CS films are usually deposited as composites, combining chitosan with inorganic and metallic particles [2,14,15,16,17,18]. The best antibacterial performance has been achieved for the CS–silver nanoparticle (AgNP) composites [16,19,20,21]. In this case, AgNPs accumulate on the bacterial membrane, thus causing bacteria cell death and the high antibacterial activity of the formed coatings [15,22]. Recently, high antibacterial activity was also reported for CS–AuNP–aminopropylsilane composites [23]. Another approach includes the introduction of metallic ions with the antibacterial effect, e.g., copper(II) ions, within the CS matrix [24].

CS-based films are typically deposited using electrophoretic deposition (EPD) [2]. This method provides a wide possibility for the process control and modification of the composition and properties of the formed films. Typical EPD is based on the neutralization mechanism proposed by Payne et al. [25]. Through this mechanism, initially dissolved CS forms a stable hydrogel on the cathode surface due to a local increase in pH caused by the oxygen reduction reaction. This technique is also useful for the deposition of CS-based composites [2,26,27]. In aqueous suspensions, protonated chitosan macromolecules can adsorb on the surface of the dispersed particles, forming positively charged core-shell structures that are then moved towards the cathode surface under an applied electrical field [2]. The main drawback of this process is that the obtained CS films are defective and porous due to the H_2_ bubbles, which are also generated on the cathode [2,28].

Different approaches have been reported to improve the quality of EPD-formed CS coatings. To date, the best results have been achieved by substituting water with alcohols [2,29]. Blanda et al. [30] utilized a galvanic method to deposit chitosan coatings on a stainless steel substrate by using a displacement reaction, with magnesium acting as a sacrificial anode. However, this method is very slow, and the formed CS films are porous.

Chitosan can effectively chelate transition metal ions, and this ability is used in wastewater-treatment applications [31]. Furthermore, a chelation-based mechanism allows one to obtain materials with a relatively uniform distribution of metallic ions in the polymer matrix [32]. Thus, their further release in bacterial media can be easily controlled [24]. Zhai et al. [33] used this ability to incorporate CS into metallic Zn layers to form composites with enhanced antibacterial activity. The chelation of CS with Cu(II) was used for the synthesis of CS–hydroxyapatite composite scaffolds with improved osteogenesis and drug-delivery ability [32]. Geng et al. [28] proposed the use of this property for the electrochemical deposition of CS films. They showed that Cu^2+^ ions in situ generated by the oxidation of a copper wire could be used to deposit a CS hydrogel with a smooth, homogeneous surface. In this case, deposition is performed at the anode of the electrochemical cell, excluding the generation of H_2_ bubbles.

Combining AgNPs with copper ions in the composition of CS-based composite films can potentially improve their antibacterial properties. The aim of this study was to develop a simple and effective method for the anodic deposition of the biomedical CS–AgNP composites based on in situ coordination with copper ions, as well as to examine their surface microstructure and antibacterial properties. To our best knowledge, this is the first attempt to combine these two approaches to obtain non-defect CS composites with enhanced antibacterial properties.

## 2. Materials and Methods

### 2.1. Materials

Chitosan powder (99.9%) of low molecular weight (50–190 kDa) was purchased from NANOSHEL and used as received. A detailed study of the physico-chemical properties of the used chitosan was reported elsewhere [34]. Acetic acid (>99.7%), trisodium citrate dihydrate (>99.0%), CuSO_4_ × 5H_2_O (>99.0%), H_2_SO_4_ (98.0%), and AgNO_3_ (>99.0%) were purchased from Chempur and used without further purification. All solutions were prepared using deionized water with a resistivity higher than 18 MΩ cm (Polwater).

### 2.2. Synthesis of AgNPs

The AgNPs used in this work were synthesized using the citrate reduction method. For this, 200 mL of a 2 mM AgNO_3_ solution were boiled in darkness, and then an excess of 1% trisodium citrate solution was added to it dropwise under continuous stirring. The formed suspension was cooled down and used as a source of AgNPs.

The size (hydrodynamic diameter) of the formed nanoparticles was examined by dynamic light scattering (DLS) using a Zetasizer ZS Malvern ZEN 3600 instrument. In these experiments, 1 µL of the formed suspension was added to 1 mL of deionized water, and then 20 scans (2 series of 10 scans each) were performed.

### 2.3. Electrodeposition of CS and CS-Based Coatings

Commercial AISI 316L stainless steel coupons with a surface area of 4 cm^2^ were used as substrates for the deposition of CS-based coatings. Before the deposition, their surface was ground with P2000 emery paper and activated in 0.1 M H_2_SO_4_.

**Figure 1:**
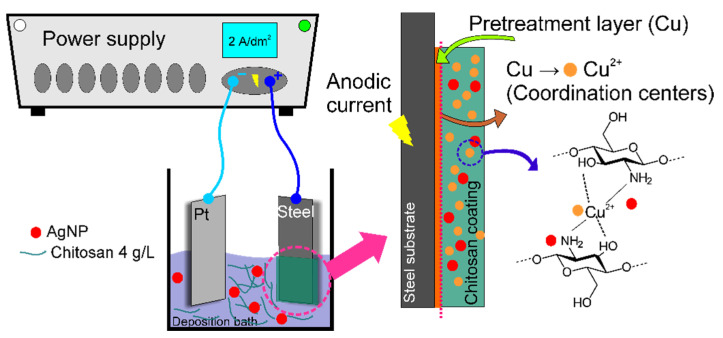
Schematic illustration of anodic CS–AgNP composite deposition using coordination with copper ions.

Figure 1 shows a schematic illustration of the used anodic coordinated deposition approach. The deposition bath was prepared by completely dissolving 4 g of CS powder in 1 L of a 1% acetic acid solution. After that, the suspension containing AgNPs was added to the formed solution in amounts of 50, 100, 150, and 200 mL/L. The pH of the formed deposition bath was adjusted to 5.5 ± 0.1 by 0.1 M NaOH. Before experiments, solutions were ultrasonically treated for 10 min.

The electrodeposition process was performed using an Autolab 302N potentiostat/galvanostat. The Cu pretreatment layer was cathodically deposited onto the steel surface from the following electrolyte [35]: 40 g/L of CuSO_4_ × 5H_2_O, 100 g/L of H_2_SO_4_, and 3 mg/L of thiourea. The deposition was performed at a cathodic current density of 2 A/dm^2^ for 25 s. A copper plate was used as the anode. After this, the surface was thoroughly rinsed with distilled water and air-dried. Such a treatment allowed us to exclude the effect of the predeposition bath components on the properties of the CS coatings. However, the addition of a small amount of sulfuric acid could enhance the stability of Ag ions [36].

The anodic coordinated deposition of CS-based coatings was performed in a 2-electrode setup with Pt foil as the cathode and steel coated with copper as the anode. The deposition was performed at a current density of 2 A/dm^2^ for 20 s. Longer deposition may result in the flaking of the formed hydrogel due to the initiation of oxygen evolution. After electrolysis, samples were removed from the deposition bath, gently rinsed with deionized water, and air-dried.

The coatings obtained in the electrolytes containing 0, 50, 100, 150, and 200 mL/L of AgNPs suspension are further labeled in the text of the present contribution as CS-0, CS-50, CS-100, CS-150, and CS-200, respectively.

### 2.4. Coatings Characterization

The morphology of the deposited CS-based coatings was examined by SEM using a JEOL JSM–7500F Field Emission Scanning Electron Microscope equipped with an EDX detector.

FT-IR spectra were measured by a Thermo Scientific Nicolet is50 Infrared Spectrometer. Spectra were recorded in a mid-infrared range (500–4000 cm^−1^) using an attenuated total reflection (ATR) accessory with a diamond crystal.

### 2.5. Antibacterial Properties

The antibacterial properties of the obtained coatings were evaluated using model reference strain of *Staphylococcus epidermidis* (*S. epidermidis*) ATCC 35984/RP62A (American Type Culture Collection). These species belong to human microbiota and can form a stable biofilm on biomedical surfaces, mainly on implants. The bacteria were inoculated on Columbia agar with 5% sheep blood (Oxoid) and incubated for 18 h at 36 °C to obtain pure cultures. Afterwards, 0.5 optical density (OD) in McFarland scale standard was made to receive a 1.5 × 10^8^ CFU (colony-forming unit) solution, and the cells’ suspension was diluted to 10^6^ CFU/mL. Then, steel samples with the deposited CS–AgNP coatings were placed on separate cell culture 12-well plates (COSTAR) and incubated for 1 and 6 h at 36 °C. Bare steel substrates were also used as a reference. After experiments, samples were removed from test plates, washed, and dried. The antibacterial performance of the coatings was evaluated using the LIVE/DEAD BacLight Bacterial Viability Kit according to the producer’s protocol. The surface analysis was performed using a ZEISS LSM780 confocal fluorescence microscope. For each sample, at least 10 images of different surface areas were taken, and the calculation of the area occupied by the life and dead bacteria was performed using the ZEN software [37]. All the measurements reported in this paper were at least triplicated.

## 3. Results and Discussion

**Figure 2:**
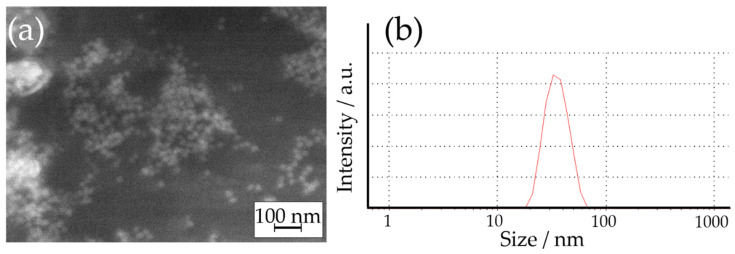
(**a**) SEM image of obtained AgNPs and (**b**) particle size distribution of AgNPs based on the DLS measurement.

At first, the synthesized dispersion of AgNPs was analyzed. Figure 2 shows the SEM image and particle size distribution of the obtained AgNPs determined by DLS. The results confirmed that spherical Ag nanoparticles were formed during the synthesis. A droplet of the dispersion of AgNPs was dried for SEM imaging, which may have caused the agglomeration of AgNPs. For this reason, the size of AgNPs was determined based on the DLS measurement that revealed their average diameter of 28 ± 4.3 nm (by number) and 31 ± 3.3 nm (by volume), which indicated their monodispersity.

**Figure 3:**
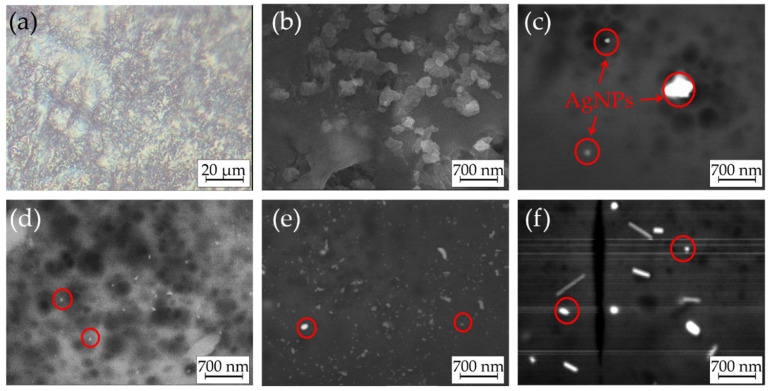
(**a**) Optical image of as-deposited and (**b**–**f**) SEM images of air-dried CS–AgNP composites deposited using coordination with copper ions: (**a**,**b**) CS-0, (**c**) CS-50, (**d**) CS-100, (**e**) CS-150, and (**f**) CS-200. Red circles in panels (**c**–**f**) indicate examples of AgNPs in the structure of CS layers.

The parameters of the electrodeposition process were adjusted during the preliminary experiments. Here, we report the results obtained using the optimized procedure. Optical and SEM images of the formed coatings are shown in Figure 3. Anodic coordinated deposition resulted in CS coatings of blue color (Figure 3a), indicating the introduction of copper(II) ions. Based on the EDX analysis, the amount of introduced Cu(II) varied in the range of 16–24 wt.% for all samples. They had a smooth surface without visible defects. Furthermore, SEM examinations of the air-dried CS–AgNP composites (Figure 3b–f) confirmed that the formed coatings did not have visible cracks even after drying. This indicated the advantages of the CS-based coatings formed using the anodic electrodeposition method, as such cracks are typical for the CS-based precipitates obtained by classical cathodic EPD [38,39,40].

The further examination of the SEM images revealed that AgNPs were successfully introduced into the coatings. The point EDX analysis from the bright surface spots (marked with red circles in Figure 3) confirmed the presence of Ag in the samples. The majority of them were introduced into the CS matrix as sub-micron aggregates that were evenly distributed over the electrode’s surface, which could be explained by partial agglomeration of AgNPs after their introduction into the deposition bath. However, a noticeable fraction of the single AgNPs with a size comparable to those observed in DLS measurements was also visible. The increase in the concentration of AgNPs in the deposition bath resulted in a higher amount of these particles in the formed coatings. Note that some microcracks were visible in the structure of the CS-200 sample. They may have been caused by internal stress occurring in the CS-based coatings upon drying [41].

**Figure 4:**
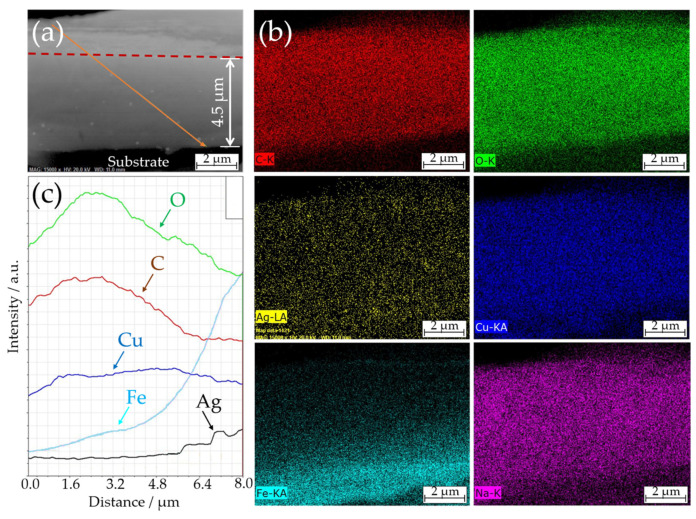
(**a**) Cross-sectional SEM image of CS-150 composite and (**b**) corresponding EDX elemental distribution maps. Panel (**c**) shows EDX elemental profiles along the solid line shown in panel (**a**).

The adhesion of the CS–AgNP coatings to the steel substrate and the distribution of the AgNPs over their thickness were examined based on the cross-sectional SEM images. An example of the cross-sectional SEM image of CS-150 composite is shown in Figure 4.

The thickness of the formed composite coatings was almost independent of the composition of the deposition bath and varied in the range of 4–5 µm. The EDX elemental distribution maps revealed a homogeneous distribution of the coating constituents through the thickness of the composite. In particular, this distribution of copper confirmed that the pre-treatment layer was fully dissolved during the anodic deposition. In turn, the slightly blurred distribution of iron at the electrode/coating interface suggested that the surface of the steel substrate might also have partially dissolved during the anodic polarization. The EDX map of Ag confirmed the impregnation of the CS matrix with AgNPs (spots of higher intensity on the EDX map). Moreover, the uniform distribution of Cu(II) ions and AgNPs suggested that the coating could potentially provide long-lasting antibacterial protection, even in the conditions of its gradual degradation in the operation media.

**Figure 5:**
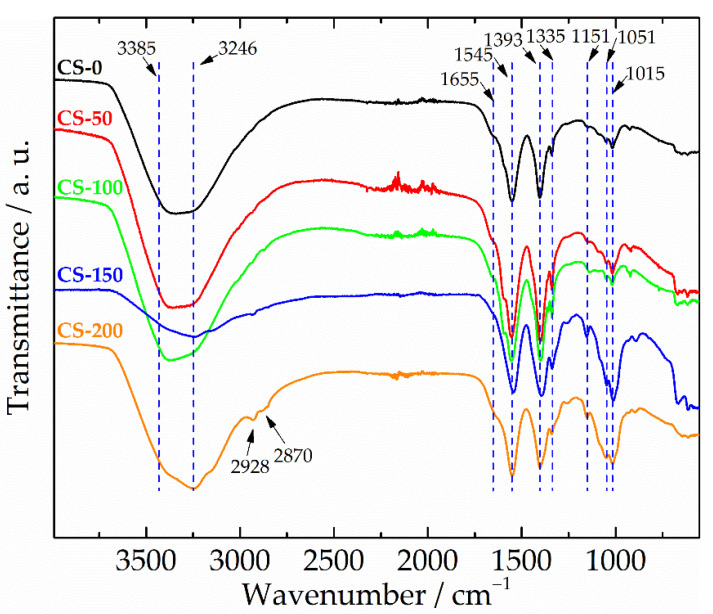
FTIR spectra of CS–AgNP composites deposited using coordination with copper ions. Spectra were normalized and are shifted on the *y*-axis for clarity.

The structure of the formed CS-based coatings was also examined by the FT-IR method; see Figure 5. In general, all samples were characterized by very similar FT-IR spectra. A strong, wide band in the region of 3385–3246 cm^−1^ corresponded to O-H stretching and N-H symmetrical vibrations originating from water and amide groups, respectively [27,39,40]. The band at 1655 cm^−1^ was the amide I band originating from the C=O and N-H stretching vibrations in the glucosamine unit. The band at 1545 cm^−1^ was attributed to the N–H bending vibrations of the amine group, known as the amide II band [40]. The peak at 1335 cm^−1^ was, probably, the amide III deformation vibration of the C–H bond linked to the N–H group. These three amide bands are usually observed in chitosan-based materials [27,39,40]. The next band at 1393 cm^−1^ was related to the –C–O– stretching of –CH_2_–OH group in chitosan [42]. The bands at 1151, 1051, and 1015 cm^−1^ originated from the vibrations of the glycosidic bonds and the C–O stretching vibration of the saccharide structure [40].

Generally, all recorded spectra were characterized by changeable and relatively low absolute intensities of the main CS-related bands, particularly in the regions of 3246–3385, 1335, and 1151–1015 cm^−1^. This could be explained by the interaction between chitosan functional groups and the deposition bath components during the anodic coordinated electrodeposition of the composites and the chelation of Cu(II) ions. Such a decrease in the relative intensity of peaks resulted in the FT-IR spectrum of the CS-200 coating showing two additional bands at 2928 and 2870 cm^−1^, attributed to the C–H stretching vibrations in the chitosan structure [40]. Similar observations were reported in the literature for composite films obtained by the electroless method [32] and cathodic EPD [39].

After analyzing the obtained results, the following mechanism of the anodic coordinated electrodeposition of the CS–AgNP composites coordinated by Cu(II) ions can be proposed. Generally, CS is insoluble in water but can be protonated in acidic media (below pH 5.5) according to the equation:

CS–NH_2_ + H^+^ → CS–NH_3_^+^ (1)

The first step in the anodic deposition mechanism can be as follows. When current passes through the system (Figure 1), the copper layer, pre-deposited onto the steel surface, starts to dissolve under the anodic polarization:

Cu → Cu^2+^ + 2e^–^ (2)

The further chelation of Cu(II) ions by CS may be explained by the Lewis acid–base theory [43]. Here, Cu(II) ions serve as the Lewis acid, accepting a pair of electrons given by the Lewis base (CS). In our case, the initial bulk pH of the deposition bath was kept around 5.5. Thus, the CS in it was only partially protonated. Unprotonated amine groups can chelate positively charged Cu(II) ions by donating the free pair of electrons from the amine nitrogen [43].

CS–NH_2_ + Cu^2+^ → CS–NH_2_Cu^2+^ (3)

On the other hand, the interaction of the protonated chitosan with Cu(II) ions may be described as follows [43,44]:

CS–NH_3_^+^ + Cu^2+^ → CS–NH_2_Cu^2+^ +H^+^ (4)

These processes result in a dense, positively charged hydrogel layer formed on the anode surface. In cases when AgNPs are present in the solution, they can electrostatically interact with the positively charged protonated CS layer and formed CS–NH_2_Cu^2+^ complexes since the zeta potential of citrate stabilized Ag nanoparticles at mildly acidic conditions is negative [45]. To summarize, the mechanism of the proposed anodic coordination deposition significantly differs from classical cathodic EPD and is based on the chelation of Cu(II) ions with CS.

**Figure 6:**
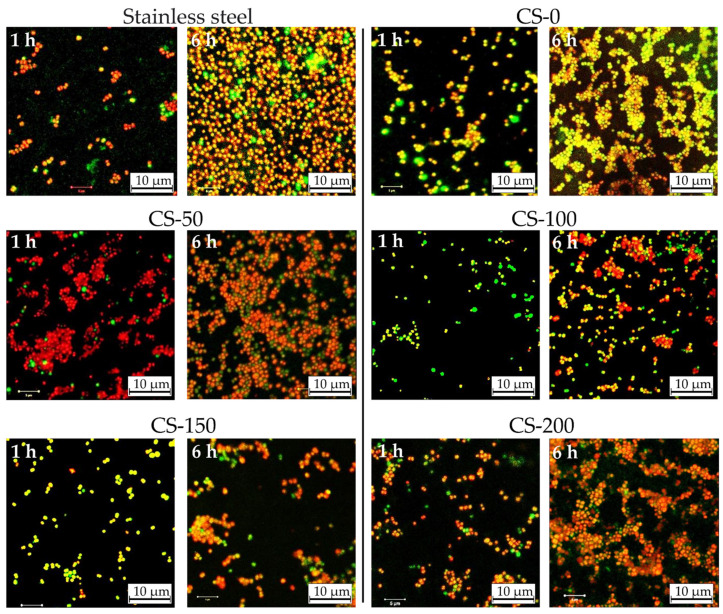
Fluorescence microscopy images of the surface of stainless steel and CS–AgNP coatings showing *S. epidermidis* ATCC 35984/RP62A bacteria with propidium iodide (red: dead bacteria) and syto9 (green: alive bacteria).

The antibacterial activity of the formed CS–AgNP coatings towards *S. epidermidis* ATCC 35984/RP62A bacteria was evaluated. Fluorescence microscope images of the surface of examined CS–AgNP composites are shown in Figure 6. It can be seen that on the surface of the reference AISI 304 samples, almost all bacteria were alive after 1 h of bacterial tests (shown in Figure 6 as green-colored colonies). After 6 h of incubation, almost the whole surface was covered with bacteria, confirming that bare steel surface could not suppress the adsorption of bacteria and formation of the biofilm. The relation of dead-to-alive bacteria cells was around 25 (Table 1).

The antibacterial activity of the CS–AgNP coatings was strongly connected to the amount of introduced AgNPs. The implementation of the anodic coordination deposition did not significantly improve the biofilm resistance of the CS-based coatings. The surface coverage of CS–AgNPs by *S. epidermidis* ATCC 35984/RP62A colonies varied in the range of 11–20% and 37–90% after 1 and 6 h of incubation, respectively. An analysis of the dead-to-live ratio of bacterial colonies revealed that the highest ratio and, correspondingly, the highest antibacterial properties had CS-150 and CS-200 coatings. This was connected to the higher concentration of AgNPs on the surface of the coatings and the release of silver ions to the bacteria medium [46]. After 6 h of tests, the highest dead-to-live ratio of 198 was attributed to the CS-200 sample, which also had the highest surface coverage by bacteria (Table 1). These data indicate that CS-based films do not possess strong anti-biofilm-forming properties, showing moderate antibacterial efficiency at high AgNP loads. The absence of a prominent antibacterial response from Cu(II) ions is probably connected to their chelation by CS. Similar observations were reported for EPD-deposited CS films [24]. In this case, the better stability of anodic CS films decreases the release rate of Cu(II) ions, thus reducing their antibacterial effectiveness. It may be expected that the antibacterial activity of the obtained films was mostly provided by the release of Ag ions from the coatings. Several mechanisms of the antibacterial activity of AgNPs have been proposed [36,47]: the inhibition of protein synthesis or a metabolic pathway, interference with cell wall synthesis, and nucleic acid synthesis. Another reported mechanism proposes the oxidation of AgNPs to Ag^+^ following the Fenton-like process, which results in the formation of active oxygen radicals [48] that destroy the membranes of the bacterial cells. Overall, the proposed anodic coordination approach is promising for the development of smart biomedical coatings and the future optimization of the relationship between the composition and antimicrobial activity of CS-based composites.

## 4. Conclusions

In the present contribution, we described a novel method for the anodic coordinated electrodeposition of CS–AgNP coatings on AISI 304 steel using in situ generated Cu(II) as the chelating agent. The proposed method allowed us to obtain crack-free smooth coatings. The results of EDX and FT-IR analyses confirmed the successful chelation of Cu(II) with CS and the introduction of AgNPs in the thickness of composites. The antibacterial effectiveness tests for *S. epidermidis* ATCC 35984/RP62A bacteria confirmed a moderate improvement in the antibacterial properties, mostly connected to the presence of AgNPs.

## Figures and Tables

**Table 1:** Viability and surface coverage of CS–AgNP coatings by *S. epidermidis* ATCC 3984/RP562A bacteria.

Sample	Average Dead-to-Live Cells Ratio		Surface Coverage by Biofilm, %	
	1 h	6 h	1 h	6 h
AISI 304 Steel	7.4	25.0	7	65
CS-0	5.8	15.3	14	72
CS-50	6.3	14.7	20	67
CS-100	9.3	6.0	12	35
CS-150	41.0	3.5	11	37
CS-200	10.4	198.0	20	90

## Data Availability

The data presented in this study are available from the corresponding authors upon reasonable request.

## References

[1] Tanpichai S., Witayakran S., Wootthikanokkhan J., Srimarut Y., Woraprayote W., Malila Y. (2020). Mechanical and antibacterial properties of the chitosan coated cellulose paper for packaging applications: Effects of molecular weight types and concentrations of chitosan. Int. J. Biol. Macromol..

[2] Avcu E., Baştan F.E., Abdullah H.Z., Rehman M.A.U., Avcu Y.Y., Boccaccini A.R. (2019). Electrophoretic deposition of chitosan-based composite coatings for biomedical applications: A review. Prog. Mater. Sci..

[3] Ibrahim A.G., Saleh A.S., Elsharma E.M., Metwally E., Siyam T. (2019). Chitosan-g-maleic acid for effective removal of copper and nickel ions from their solutions. Int. J. Biol. Macromol..

[4] Liu M., Zhou Y., Zhang Y., Yu C., Cao S. (2014). Physicochemical, mechanical and thermal properties of chitosan films with and without sorbitol. Int. J. Biol. Macromol..

[5] Marangoni Júnior L., Vieira R.P., Jamróz E., Anjos C.A.R. (2021). Furcellaran: An innovative biopolymer in the production of films and coatings. Carbohydr. Polym..

[6] Hu J., Zhu J., Ge S., Jiang C., Guo T., Peng T., Huang T., Xie L. (2020). Biocompatible, hydrophobic and resilience graphene/chitosan composite aerogel for efficient oil−water separation. Surf. Coat. Technol..

[7] Li W.W., Wang H.Y., Zhang Y.Q. (2017). A novel chitosan hydrogel membrane by an improved electrophoretic deposition and its characteristics in vitro and in vivo. Mater. Sci. Eng. C.

[8] Yilmaz Atay H., Jana S., Jana S. (2019). Functional Chitosan.

[9] Ali A., Ahmed S. (2018). A review on chitosan and its nanocomposites in drug delivery. Int. J. Biol. Macromol..

[10] Kong M., Chen X.G., Xing K., Park H.J. (2010). Antimicrobial properties of chitosan and mode of action: A state of the art review. Int. J. Food Microbiol..

[11] Rabea E.I., Badawy M.E.T., Stevens C.V., Smagghe G., Steurbaut W. (2003). Chitosan as Antimicrobial Agent: Applications and Mode of Action. Biomacromolecules.

[12] Foster L.J.R., Butt J. (2011). Chitosan films are NOT antimicrobial. Biotechnol. Lett..

[13] Qi L., Xu Z., Jiang X., Hu C., Zou X. (2004). Preparation and antibacterial activity of chitosan nanoparticles. Carbohydr. Res..

[14] Rozmysłowska-Wojciechowska A., Karwowska E., Gloc M., Woźniak J., Petrus M., Przybyszewski B., Wojciechowski T., Jastrzębska A.M. (2020). Controlling the Porosity and Biocidal Properties of the Chitosan-Hyaluronate Matrix Hydrogel Nanocomposites by the Addition of 2D Ti3C2Tx MXene. Materials.

[15] Li J., Zhuang S. (2020). Antibacterial activity of chitosan and its derivatives and their interaction mechanism with bacteria: Current state and perspectives. Eur. Polym. J..

[16] Cometa S., Bonifacio M.A., Baruzzi F., de Candia S., Giangregorio M.M., Giannossa L.C., Dicarlo M., Mattioli-Belmonte M., Sabbatini L., De Giglio E. (2017). Silver-loaded chitosan coating as an integrated approach to face titanium implant-associated infections: Analytical characterization and biological activity. Anal. Bioanal. Chem..

[17] Titov V., Nikitin D., Naumova I., Losev N., Lipatova I., Kosterin D., Pleskunov P., Perekrestov R., Sirotkin N., Khlyustova A. (2020). Dual-Mode Solution Plasma Processing for the Production of Chitosan/Ag Composites with the Antibacterial Effect. Materials.

[18] Ciraldo F., Schnepf K., Goldmann W., Boccaccini A. (2019). Development and Characterization of Bioactive Glass Containing Composite Coatings with Ion Releasing Function for Antibiotic-Free Antibacterial Surgical Sutures. Materials.

[19] Arjunan N., Singaravelu C.M., Kulanthaivel J., Kandasamy J. (2017). A potential photocatalytic, antimicrobial and anticancer activity of chitosan-copper nanocomposite. Int. J. Biol. Macromol..

[20] Kumar-Krishnan S., Prokhorov E., Hernández-Iturriaga M., Mota-Morales J.D., Vázquez-Lepe M., Kovalenko Y., Sanchez I.C., Luna-Bárcenas G. (2015). Chitosan/silver nanocomposites: Synergistic antibacterial action of silver nanoparticles and silver ions. Eur. Polym. J..

[21] Sanpui P., Murugadoss A., Prasad P., Ghosh S., Chattopadhyay A. (2008). The antibacterial properties of a novel chitosan–Ag-nanoparticle composite. Int. J. Food Microbiol..

[22] Raghavendra G.M., Jung J., Kim D., Seo J. (2016). Microwave assisted antibacterial chitosan–silver nanocomposite films. Int. J. Biol. Macromol..

[23] Virgili A.H., Laranja D.C., Malheiros P.S., Pereira M.B., Costa T.M.H., de Menezes E.W. (2021). Nanocomposite film with antimicrobial activity based on gold nanoparticles, chitosan and aminopropylsilane. Surf. Coat. Technol..

[24] Akhtar M.A., Ilyas K., Dlouhý I., Siska F., Boccaccini A.R. (2020). Electrophoretic Deposition of Copper(II)–Chitosan Complexes for Antibacterial Coatings. Int. J. Mol. Sci..

[25] Liu Y., Kim E., Ghodssi R., Rubloff G.W., Culver J.N., Bentley W.E., Payne G.F. (2010). Biofabrication to build the biology-device interface. Biofabrication.

[26] Saleem O., Wahaj M., Akhtar M.A., Ur Rehman M.A. (2020). Fabrication and Characterization of Ag–Sr-Substituted Hydroxyapatite/Chitosan Coatings Deposited via Electrophoretic Deposition: A Design of Experiment Study. ACS Omega.

[27] Nawrotek K., Grams J. (2021). Understanding Electrodeposition of Chitosan–Hydroxyapatite Structures for Regeneration of Tubular-Shaped Tissues and Organs. Materials.

[28] Geng Z., Wang X., Guo X., Zhang Z., Chen Y., Wang Y. (2016). Electrodeposition of chitosan based on coordination with metal ions: In situ-generated by electrochemical oxidation. J. Mater. Chem. B.

[29] Mahmoodi S., Sorkhi L., Farrokhi-Rad M., Shahrabi T. (2013). Electrophoretic deposition of hydroxyapatite–chitosan nanocomposite coatings in different alcohols. Surf. Coat. Technol..

[30] Blanda G., Brucato V., Carfì F., Conoscenti G., La Carrubba V., Piazza S., Sunseri C., Inguanta R. (2019). Chitosan-Coating Deposition via Galvanic Coupling. ACS Biomater. Sci. Eng..

[31] Gamage A., Shahidi F. (2007). Use of chitosan for the removal of metal ion contaminants and proteins from water. Food Chem..

[32] Gritsch L., Maqbool M., Mouriño V., Ciraldo F.E., Cresswell M., Jackson P.R., Lovell C., Boccaccini A.R. (2019). Chitosan/hydroxyapatite composite bone tissue engineering scaffolds with dual and decoupled therapeutic ion delivery: Copper and strontium. J. Mater. Chem. B.

[33] Zhai X., Sun C., Li K., Guan F., Liu X., Duan J., Hou B. (2016). Synthesis and characterization of chitosan–zinc composite electrodeposits with enhanced antibacterial properties. RSC Adv..

[34] Santini E., Jarek E., Ravera F., Liggieri L., Warszynski P., Krzan M. (2019). Surface properties and foamability of saponin and saponin-chitosan systems. Colloids Surf. B Biointerfaces.

[35] Kasach A.A., Kharitonov D.S., Makarova I.V., Wrzesińska A., Zharskii I.M., Kurilo I.I. (2020). Effect of thiourea on electrocrystallization of Cu–Sn alloys from sulphate electrolytes. Surf. Coat. Technol..

[36] Mofidfar M., Kim E.S., Larkin E.L., Long L., Jennings W.D., Ahadian S., Ghannoum M.A., Wnek G.E. (2019). Antimicrobial Activity of Silver Containing Crosslinked Poly(Acrylic Acid) Fibers. Micromachines.

[37] Kruk T., Gołda-Cępa M., Szczepanowicz K., Szyk-Warszyńska L., Brzychczy-Włoch M., Kotarba A., Warszyński P. (2019). Nanocomposite multifunctional polyelectrolyte thin films with copper nanoparticles as the antimicrobial coatings. Colloids Surf. B Biointerfaces.

[38] Oliveira J.A.M., de Santana R.A.C., de OliveiraWanderley Neto A. (2021). Electrophoretic deposition and characterization of chitosan-molybdenum composite coatings. Carbohydr. Polym..

[39] Oliveira J.A.M., de Santana R.A.C., de OliveiraWanderley Neto A. (2020). Characterization of the chitosan-tungsten composite coating obtained by electrophoretic deposition. Prog. Org. Coat..

[40] Gebhardt F., Seuss S., Turhan M.C., Hornberger H., Virtanen S., Boccaccini A.R. (2012). Characterization of electrophoretic chitosan coatings on stainless steel. Mater. Lett..

[41] Cordero-Arias L., Cabanas-Polo S., Gao H., Gilabert J., Sanchez E., Roether J.A., Schubert D.W., Virtanen S., Boccaccini A.R. (2013). Electrophoretic deposition of nanostructured-TiO2/chitosan composite coatings on stainless steel. RSC Adv..

[42] Sun F., Pang X., Zhitomirsky I. (2009). Electrophoretic Deposition of Composite Hydroxyapatite–Chitosan–Heparin Coatings. J. Mater. Process. Technol..

[43] Rhazi M., Desbrières J., Tolaimate A., Rinaudo M., Vottero P., Alagui A. (2002). Contribution to the study of the complexation of copper by chitosan and oligomers. Polymer.

[44] Vold I.M.N., Vårum K.M., Guibal E., Smidsrød O. (2003). Binding of ions to chitosan—selectivity studies. Carbohydr. Polym..

[45] Bhattarai N., Khanal S., Pudasaini P.R., Pahl S., Romero-Urbina D. (2011). Citrate Stabilized Silver Nanoparticles. Int. J. Nanotechnol. Mol. Comput..

[46] Ryan C., Alcock E., Buttimer F., Schmidt M., Clarke D., Pemble M., Bardosova M. (2017). Synthesis and Characterisation of Cross-Linked Chitosan Composites Functionalised with Silver and Gold Nanoparticles for Antimicrobial Applications. Sci. Technol. Adv. Mater..

[47] Guzman M., Dille J., Godet S. (2012). Synthesis and Antibacterial Activity of Silver Nanoparticles against Gram-Positive and Gram-Negative Bacteria. Nanomed. Nanotechnol. Biol. Med..

[48] He D., Miller C.J., Waite T.D. (2014). Fenton-like zero-valent silver nanoparticle-mediated hydroxyl radical production. J. Catal..

